# Supplementary material for: Genetic Characterization of Two Novel Insect-Infecting Negative-Sense RNA Viruses Identified in a Leaf Beetle, Aulacophora indica
Source: Insects. 2024 Aug 15;15(8):615. doi: 10.3390/insects15080615 (PMC11354625; doi:10.3390/insects15080615)
Supplement: Supplementary file 1 [file insects-15-00615-s001.zip › Table S1. Primers used in this study.pdf]

Table S1. Primers used in this study.

Primer	Sequence (5' to 3')	Purpose
Long primer	CTAATACGACTCACTATAGGGCAAGCAGTGG TATCAACGCAGAGT	Amplification of 5′/3′ RACE fragment
Short primer	CTAATACGACTCACTATAGGGC	
5′-RACE GSP	CGCCATGACATCGCACGACCTAAGTCC	Amplification of 5′ RACE fragment of AINLV1
3′-RACE GSP	CTTGATGGGACCCTCACCACTGCCAC	Amplification of 3′ RACE fragment of AINLV1
AINLV1-1F	AGAGATGACGACTGTGGTGGAA	Amplification of AINLV1 viral genome
AINLV1-1R	GGTGTGGAGGTGGTGGCATA	
AINLV1-2F	ACAACCAACCTCAACAACAACT	
AINLV1-2R	CGCCACCGCCACTATTATTAG	
AINLV1-3F	GACGAAACTATGTATTCAGACC	
AINLV1-3R	GTCACATGGTAGACTCTCTTCG	
AINLV1-4F	GAGATGACGACTGTGGTGGAA	
AINLV1-4R	CGGAGTGGCGAATCATTGAC	
AINLV1-5F	GACTCCTGCTCTATTCCTTGG	

AINLV1-5R	TGAACTTCGTGACTACCTCTAC
AINLV1-6F	GCGCAAACAAATACT
AINLV1-6R	CGTCTTGAATCTGAGGGCC
AINLV1-7F	GCCCTCAGATTCAAGACGAACT
AINLV1-7R	GTACTGGTGTTGCCTGCTCTC
AINLV1-8F	CCAGCCAAGGAATCTACATCAG
AINLV1-8R	CGAAGCTCATACCTCTCCAGTA
AINLV1-9F	ATCCATCTGCCTTACGCACATC
AINLV1-9R	CCAGGACATCGCTAGGAGACA
AINLV1-10F	GGTGCTACTCTGTTGGAGGTAT
AINLV1-10R	ACGAGGATGTAAGTGAGTGTCT
AINLV1-11F	CATTCTCCTGGGACCTTTGAC
AINLV1-11R	CAGATGAGAGATGCCTGTCTTTG
AINLV1-12F	CCCAAGATACCCGACTTGTTGA
AINLV1-12R	GGTTGATGCTGGAGACGACAT

AINLV1-13F	CGATATGGATGCGAGTGTCAAG	
AINLV1-13R	AAGGTGTGGCAAGCTCTAATCT	
AINLV1-14F	CTCGCCAGAACAATATCCAGGA	
AINLV1-14R	CCGCAGCCACTTCATCTTCC	
AINLV1-15F	GCAAACCAGACTTTACCAACCA	
AINLV1-15R	CGCATAAGCAAGCAAGGTCAT	
AINLV1-16F	TGTGGGCAAACGAGAAAGAACT	
AINLV1-16R	AAGCCTAGCAGCGGTGAGT	
AINLV1-17F	GCACACGTATCATTCCGAAGG	
AINLV1-17R	GGCAGATGGTTGGCAAATAACA	
AINLV1-18F	GCATATTACCGAGCCACCTG	
AINLV1-18R	GACCAGCCAGACTCTAGATTCA	
AICLV1-1F	GGTAATCACACGTAGCAAAGC	
AICLV1-1R	GGTCCATGTTGACGATAGGG	Amplification of AICLV1 viral genome
AICLV1-2F	GGTAATCACACGTAGCAAAGC	

AICLV1-2R	CCGATCATCTATGAATGTCG
AICLV1-3F	CCTCCGCATCTTCCGCAAT
AICLV1-3R	GCTCCTTCGTCCTTCTCTAACT
AICLV1-4F	GCAATGGCTGAACAAGAACAAG
AICLV1-4R	AATAGGACACGGATGAGGACAA
AICLV1-5F	ACTAGACTCCTGCTCGCCTATC
AICLV1-5R	CTCCAAGATGCTTCGTCCTGAT
AICLV1-6F	CACTCTCGACGAGTTATCAG
AICLV1-6R	GCTTTGCTACGTGTGATTACC
AICLV1-7F	AGAACCTGCCTTAGAAGCCTTA
AICLV1-7R	CGCCTAGAAGCAATTTAATCCC
AICLV1-8F	CTCCAACTGCTCAATTCGTCAA
AICLV1-8R	GCCTGACCAACAAGTGTTCATC
AICLV1-9F	TCACTTGGCAGACTCCTCAA
AICLV1-9R	TTCCATCCAGTAACTCCTTGTT

AICLV1-10F	TCAATTACCCTGACAACGCTTA
AICLV1-10R	AACTGCATGACGTTCTTCTCTT
AICLV1-11F	AAAGTCATCAGATGCACAGTCT
AICLV1-11R	AATGGCATTAGTTCGGCTACTG
AICLV1-12F	ACACTGACACCTAATGTCCACT
AICLV1-12R	AACCGATCCTGAAGCCTAATCT
AICLV1-13F	CCACCCAATGAACAAACAGGAC
AICLV1-13R	GCAGAGTATCACCAGAGCACAC

Abbreviations: GSP, Gene Specific Primer; AINLV1, Aulacophora indica Nyami-like virus 1; AICLV1, Aulacophora indica chu-like virus 1.
